# Genome-Wide Identification, Function, and Expression Analysis of the ABC Transporter Gene Family in Forest Musk Deer (*Moschus berezovskii*) Under Musk Secretion Stage

**DOI:** 10.3390/ani15243630

**Published:** 2025-12-17

**Authors:** Ying-Ying Ren, Xuan-Ze Zhou, Jin-Fang Ma, Xue-Mei Jiang, Fang Dan, Dan-Dan Liao, Cong-Xue Yao, Cheng-Li Zheng, Wen-Hua Qi

**Affiliations:** 1 Three Gorges Reservoir Area Environment and Ecology of Chongqing Observation and Research Station, College of Environmental and Chemical Engineering, Chongqing Three Gorges University, Chongqing 404100, China; 2 College of Biological and Food Engineering, Chongqing Three Gorges University, Chongqing 404100, China; 3 Chongqing Three Gorges Academy of Agricultural Sciences, Chongqing Three Gorges University, Chongqing 404100, China; 4 Beijing Yanshe Biotechnology Development Co., Ltd., Beijing 102200, China; 5 Sichuan Institute of Musk Deer Breeding, Chengdu 611830, China

**Keywords:** *Moschus berezovskii*, ATP-binding cassette (ABC) transporter, genome-wide identification, expression

## Abstract

**Simple Summary:**

The forest musk deer (FMD, *Moschus berezovskii*) is a small mammal belonging to the genus Moschus within the family Moschidae, renowned for the precious musk secreted by the musk glands of its males. However, research into the expression of gene family members within the musk gland tissue and acinar cells of the FMD remains limited. *ABC* transporters play a crucial role in substance transport within animal and plant organisms. This study identifies 51 *ABC* genes for the first time in the FMD. Preliminary validation was conducted using transcriptome sequencing data from FMD musk gland tissue, followed by RT-qPCR validation using cells from in vitro cultured musk gland tissue. These findings lay the groundwork for subsequent functional genomics research.

**Abstract:**

The ATP-binding cassette (ABC) transporter family is one of the oldest conserved protein families and is widely present in animal and plant cells. However, few studies have investigated the role of ABC in the forest musk deer (FMD; *Moschus berezovskii*). In this study, we employed bioinformatics methods to identify and analyze the ABC transporter genes in *M. berezovskii* to elucidate the potential function of ABC genes in musk secretion. A total of 51 members of the MbABC gene family were identified. The analysis encompassed various aspects including physical and chemical properties, phylogenetic tree, structure prediction, conserved motifs, gene structures, chromosome localization, collinearity analysis, and KEGG and GO enrichment. Collinearity analysis revealed that the ABC transporter gene family is conserved in FMD, Cervidae, and five Bovinae species. *MbABCB6*, *MbABCD4*, *MbABCF3*, and *MbABCG5* are key genes in protein–protein interaction networks, which are primarily involved in the transport of vitamins, lipids, and proteins. Tissue expression analysis showed that MbABCs were expressed at different stages. The RT-qPCR analysis revealed that 12 *MbABC* genes were up-regulated in musk gland cells during the non-secretion phase and stimulation phase, particularly *MbABCC4d* and *MbABCC3*. This study provides comprehensive information on the *ABC* gene family in FMD which can be further used for their functional validation.

## 1. Introduction

The ATP-binding cassette (ABC) family represents one of the most ancient and evolutionarily conserved membrane transporter systems, present in both eukaryotes and prokaryotes [1]. These proteins are located in various cellular membranes, including the plasma membrane, endoplasmic reticulum, and Golgi apparatus [2], where they function as ATP-driven transporters facilitating substrate translocation and modulating diverse cellular processes [3]. Structurally, ABC proteins consist of transmembrane domain (TMD) and nucleotide-binding domain (NBD). The classic ABC transporter comprises two TMDs and two NBDs [4]. The NBDs are highly conserved across the family and are responsible for ATP binding and hydrolysis, whereas the TMDs—which form substrate translocation pathways—exhibit considerable sequence divergence, enabling the transport of a broad spectrum of substrates such as ions, lipids, hormones, xenobiotics, secondary metabolites, and reactive oxygen species (ROS)-related compounds [5]. Based on domain architecture, ABC transporters are categorized into three types: full-size transporters (two TMDs and two NBDs), half-size transporters (one TMD and one NBD, which dimerize to form functional units), and soluble transporters (lacking TMDs and involve in non-transport roles) [6,7]. Phylogenetic analyses based on NBD sequence homology have led to the classification of ABC transporters into eight subfamilies: ABCA-ABCH [8]. Among these, ABCA–ABCG are present in animal genomes, such as human (*Homo sapiens*) [9], with ABCB, ABCC, and ABCG being the most extensively characterized [10]. In contrast, the ABCH subfamily has been identified only in specific animal lineages, such as fruit fly (*Drosophila melanogaster*) [11] and zebrafish (*Danio rerio*) [12].

In mammals, ABCA subfamily members are primarily involved lipid transport [13], whereas ABCB transporters contribute to detoxification by effluxing endogenous metabolites and xenobiotics [14]. The ABCC subfamily influences pharmacokinetics through ATP-dependent transport of various compounds [15], and ABCD proteins are implicated in fatty acid transport and metabolism via intrinsic thioesterase activity [16]. ABCF proteins participate in translational regulation, ribosome assembly, and protein synthesis [17], while ABCG transporters, expressed in tissues such as the liver and intestine, regulate sterol transport—including cholesterol—and mediate drug efflux [18,19].

The forest musk deer (FMD; *Moschus berezovskii*) is an artiodactyl mammal primarily distributed in Asia. Musk is a precious Chinese herbal remedy and a premium raw ingredient [20]. FMD have musk glands between their navels and genitals that secrete musk [21], valued for its secretion of musk—a substance widely used in traditional medicine and perfumery due to its distinctive fragrance, anti-inflammatory and antitumor properties, and effects on the central nervous and cardiovascular systems [22]. The principal chemical constituents of musk comprise various proteins and peptides, alongside macrocyclic and steroidal [23,24,25]. However, wild FMD populations have experienced a severe decline in recent decades, driven by overexploitation, habitat destruction, and degradation [26], leading to their classification on the International Union for Conservation of Nature (IUCN) Red List of Threatened Species. Captive breeding programs initiated in China since the 1950s have not sufficiently mitigated population decline or met the high market demand for musk [27]. Consequently, a deeper understanding of the molecular mechanisms underlying musk secretion is urgently needed to inform strategies for enhancing musk production [28].

To date, comprehensive genomic surveys of *ABC* transports have been conducted in various vertebrates, including sea lamprey (*Petromyzon marinus*) and Japanese lamprey (*Lethenteron camtschaticum*) [29], Common Carp (*Cyprinus carpio*) [30], mouse (*Mus musculus*) [31], and human [32,33]. However, a systematic analysis of the ABC transport gene family in FMD has not yet been reported, and lipids constituted 86–88% of natural musk [23]. Consistent with this, a study by Liu et al. revealed significant enrichment of pathways related to ‘fatty acid metabolism’ and ‘unsaturated fatty acid biosynthesis’ in the cells [34]. Since ABC transporters are functionally implicated in the transport of lipids and steroids, it is, therefore, plausible that ABC genes fulfil a vital function in the musk secretion stage. We hypothesize that high expression of these genes facilitates and enhances musk secretion in FMD. Therefore, in this study, we performed a genome-wide identification and characterization of ABC transporter gene family in a chromosome-level genome assembly of FMD. We analyzed their physicochemical properties, gene structure, chromosomal localization, phylogenetic tree, collinearity analysis, KEGG and Gene Ontology (GO) enrichment, and protein–protein interaction (PPI) networks. Furthermore, using RNA-seq analysis and RT-qPCR validation, we investigated the expression profiles of 14 *MbABC* genes in the musk gland cells under agonist-stimulated and non-stimulated conditions, simulating the secretory phase of musk production. This study provides foundational insights into the molecular mechanisms of musk secretion and supports conservation-oriented applications for sustainable musk production.

## 2. Materials and Methods

### 2.1. Genome-Wide Identification of MbABC Family Genes

The chromosome-level genome and annotation of FMD was downloaded from MuskDB (http://muskdb.cn/home/, accessed on 11 April 2024). The protein sequence of *H. sapiens*, *M. musculus*, domestic cattle (*Bos taurus*), goat (*Capra hircus*), sheep (*Ovis aries*), and zebrafish (*Danio rerio*) were downloaded from UniProt database (https://www.uniprot.org/, accessed on 5 May 2025). Subsequently, blastP (E-value < 1 × 10^−5^) was performed using FMD’s whole-genome proteome to obtain candidate protein sequences. In order to identify TMD and NBD domains, the program Pfam (http://pfam-legacy.xfam.org/, accessed on 8 May 2025) was used to identify the ABC gene family of the FMD [35]. These models were then utilized within the TBtools software (version 2.082) [36] to screen for ABC transporters in FMD. Finally, the conserved domains of all identified protein sequences were confirmed using the CDD database [37] and SMART technology [38] to further screen and identify members of the ABC transporters gene family in FMD.

### 2.2. Physical and Chemical Properties Analysis of MbABC Proteins

The ExPASy ProtParam (https://web.expasy.org/protparam/, accessed on 12 May 2025) was used to determine the physicochemical properties. To examine the signal peptides, we used SignalP-4.1 Server (https://services.healthtech.dtu.dk/services/SignalP-4.1/, accessed on 13 May 2025), while subcellular localization was predicted using the WoLF PSORT (https://wolfpsort.hgc.jp/, accessed on 13 May 2025).

### 2.3. Phylogenetic Tree Analysis of MbABC Proteins

All available ABC transporter protein sequences of 40 *B. taurus*, 42 *Ca. hircus*, and 48 *O. aries* were retrieved from the NCBI database (https://www.ncbi.nlm.nih.gov/, accessed on 25 November 2025) and used for the phylogenetic analyses. The protein sequences were used in the phylogenetic tree which was analyzed using the Maximum Likelihood (ML) method based on bootstrap sampling in MEGA software (version 12), with a bootstrap method of 1000. The parameters were as follows: Jones–Taylor–Thornton (JTT) model; Gamma Distributed (G); partial deletion 95%; 1000 bootstrap replications. The phylogenetic tree was visualized using the Interactive Tree of Life (iToL) (https://itol.embl.de/, accessed on 30 November 2025).

### 2.4. Structure Prediction of MbABC Proteins

The secondary structure of MbABC gene protein was predicted using the PRABI-GERLAND (https://npsa.lyon.inserm.fr/cgi-bin/npsa_automat.pl?page=/NPSA/npsa_sopma.html, accessed on 14 May 2025). The tertiary structure of the MbABC genes protein using the SWISS-MODEL to visualize (https://swissmodel.expasy.org/, accessed on 16 May 2025) [39].

### 2.5. Conserved Motifs and Gene Structures Domain Analysis of MbABC Proteins

The conserved motifs of MbABC proteins were predicted using MEME, setting the maximum modulus value to 8 and the rest of the parameters to default values (http://meme-suite.org/tools/meme, accessed on 25 May 2025). The meme prediction results of the sequences, the gene structure and the structure with were integrated and visualized using TBtools software.

### 2.6. Chromosome Localization and Collinearity Analysis of MbABC Proteins

Based on the whole genome annotation files, chromosomal localization information of ABC gene family was extracted, including gene ID, chromosomal location and corresponding chromosome length. Subsequently, TBtools was used to visualize and present the physical distribution of ABC genes on *M. berezovskii* chromosomes.

The whole genome sequence of domestic cattle, sheep, goat, water buffalo, yak, and red deer were obtained from the Ensembl Ftp database (https://ftp.ensembl.org/, accessed on 3 June 2025). TBtools was used to obtain the collinearity relationships among FMD, *B. taurus*, *O. aries*, *Ca. hircus*, *Bu. bubalis*, *B. grunniens*, and *Ce. elaphus*. The syntenic and homology relationships between the studied species were analyzed and visualized using TBtools.

### 2.7. KEGG and GO Enrichment Analysis of MbABC Proteins

The protein sequences of *MbABC* genes family were annotated on EGGNOG-mapper database (http://eggnog6.embl.de/, accessed on 8 June 2025). The selected MbABC genes family members were enriched in GO and KEGG database. The threshold for significant enrichment is set to *p* < 0.05.

### 2.8. Protein–Protein Interaction Network Analysis of MbABC Proteins

To investigate protein–protein interactions, we used the String online database (https://cn.string-db.org/, accessed on 8 June 2025). The protein–protein interaction network was visualized using Cytoscape software (version 3.10.3).

### 2.9. Analysis of the Expression of ABCs in Different Stages and Tissues of MbABC Proteins

Musk gland tissues from different developmental stages of the forest musk deer were sent for standard transcriptome sequencing (detailed protocol is provided in the Supplementary Materials). We utilized TBtool to extract the expression levels of *MbABC* genes across four conditions: the peak secretion phase, late secretion phase, muscle tissue, and adult musk gland tissue. The FPKM values were used to analyzed for gene expression level. The data were then analyzed using one-way analysis of variance (ANOVA) in SPSS (version 26), with a significance threshold of *p* < 0.05. Finally, the results were visualized using GraphPad Prism software (version 10.1.2).

### 2.10. RT-qPCR Analysis of MbABC Proteins

To evaluate the biological function of the MbABC gene, in vitro-adapted forest musk gland cells were cultured in two phases: non-secretory and stimulated secretion. The musk gland cells and agonist were purchased from Beijing Yanshe Biotechnology Development Co., Ltd. (Beijing, China). The company indicated that adding this agonist to the culture medium would advance the musk gland cells from the non-secretory phase to the secretory phase. First, primary forest musk gland cells were cultured at incubator with 37 °C, 5% CO_2_. Upon achieving stability after two passages, the cells were segregated into two experimental groups: a control group and an agonist-stimulated group, each consisting of six 225 cm^2^ culture flasks. At a confluence of approximately 80%, corresponding to a cell density of 1 × 10^6^ per flask, the respective treatments were administered. The control group was cultured in unchanged medium for 24 h, whereas the stimulated group received fresh medium containing the agonist for the same duration, after which cells were harvested for RNA extraction.

Total RNA was extracted strictly according to the GOONIE Cell/Tissue RNA Extraction Kit (Cat#400-105, Guangdong, China), with concentration determined via micro-spectrophotometry. Reverse transcription was performed using the GOONIE Fast First-Strand cDNA Synthesis Mix for RT (with dsDNase) kit (Cat#500-101). RT-qPCR was conducted using the GOONIE Fast Tap SYBR Green qPCR Mix kit (Cat#500-100). The RT-qPCR mixture contained 2 μL cDNA, 10 μL SYBR Green Fast Tap Mix, 0.4 μL of each primer, and 7.2 μL nuclease-free water. The reaction was cycled at 95 °C for 30 s, followed by 40 cycles of 95 °C for 10 s and 60 °C for 30 s. Perform one melting curve cycle run at 95 °C. All temperatures and durations were set to instrument defaults. The fragments were amplified using specific primers that were designed using an online toolkit from the NCBI database (http://www.ncbi.nlm.nih.gov/tools/primer-blast/, accessed on 16 August 2025). The 2^−∆∆Ct^ method [40] was applied to calculate the relative expression levels of MbABCs, with three biological replicates for each treatment. The mean ± SE of three tests was presented in the data. Three biological replicates were used for each experiment, and differences in data between the two groups used for comparison were rated as statistically significant (* *p* < 0.05) or extremely significant (**** *p* < 0.0001). The primers for RT-qPCR assays are shown in Table S1.

## 3. Results

### 3.1. Identification and Chromosomal Localization Analysis of MbABC Proteins

**Figure 1:**
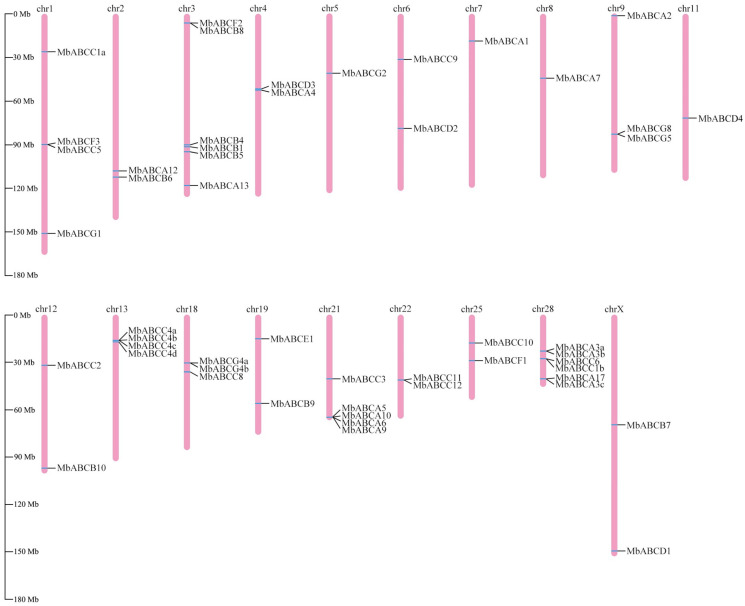
Chromosomal location distribution of MbABC proteins.

In this study, we conducted a whole-genome analysis to identify members of the ABC transporter family in FMD, combined with the conserved domain information predicted by NCBI CDD and Pfam, sequences lacking annotation information and conserved domains were excluded. At the whole-genome level, 51 ABC genes were identified in FMD. These genes were distributed across seven subfamilies (ABCA-ABCG). Chromosome mapping of these genes revealed an uneven distribution across 19 chromosomes in FMD (Figure 1). Some genes from the ABCA, ABCC, and ABCG subfamilies may have undergone tandem duplication during evolution. The *ABC* protein-encoding sequences demonstrated significant differences, with CDs lengths ranging from 7296 to 393,457 bp. Based on protein domains, they are classified into 27 full-size transporters, 21 half-size transporters, and three soluble transporters (Table S2).

### 3.2. Physicochemical Analysis of MbABC Proteins

The physicochemical properties analysis of *ABC* transporter proteins in FMD is provided (Table S3). There are significant differences in the physicochemical properties among family members. The ABC protein with amino acid lengths ranging from 569 to 5246 residues, with members exceeding 1000aa accounting for 60.1% of the total. The molecular weights range from 63 to 590 kDa. Furthermore, the theoretical isoelectric point (pI) of the ABC proteins ranges from 5.54 to 9.84, with 32 members having a pI greater than 7, representing 62.7% of the total. The stability of a protein is indicated by its instability index, where a value less than 40 suggests that the protein is stable; whereas a value greater than 40 implies that the protein may be unstable. Within the proteins, 56.8% of the members are stable proteins, and 72.5% are hydrophobic proteins. Notably, subcellular localization analysis revealed that the ABC proteins were primarily located in the plasma membrane, nucleus, cytoplasm, and mitochondria.

### 3.3. Secondary Structure and Tertiary Structure Models of MbABC Proteins

**Figure 2:**
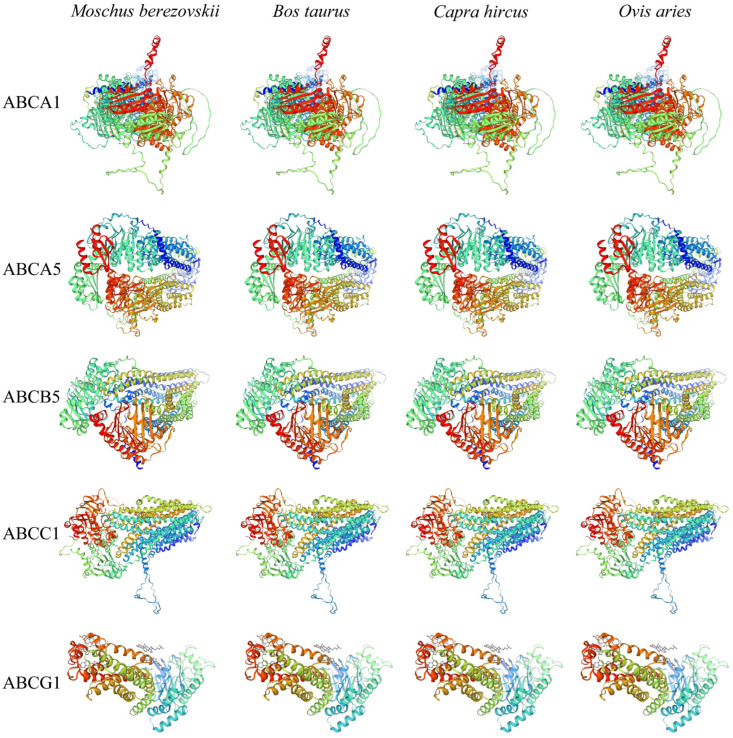
Predictive structure of *ABC* proteins in FMD, *B. taurus*, *O. aries*, and *Ca. hircus*.

The secondary structure prediction (Table S4) indicates that alpha helix structure accounts for the largest proportion, ranging from 62.03% (*MbABCA13*) to 38.73% (*MbABCE1*). The second most abundant structure is the random coil, with a proportion ranging from 41.41% (*MbABCG4b*) to 23.57% (*MbABCB9*). This indicates that alpha helix and random coils play a major role in the formation of the tertiary structure of FMD. Homology modeling is an important technique in structural biology [39]. To gain insight into the structural characteristics of the *MbABC* proteins, we selected one protein from each group of FMD, cattle, goat, and sheep. Then, we generated a three-dimensional protein model using SWISS-MODEL. The results showed that *ABCA1*, *ABCA5*, *ABCB5*, *ABCC1*, and *ABCG1* proteins had similar structures in different species (Figure 2). Collectively, the proteins from different species within the same subfamily exhibited considerable differences, whereas those from different subfamilies within the same species exhibited considerable differences. This result revealed the structural diversity of the ABC family in the four species. The study suggests that the ABC transporter family exhibits structural diversity among different species, further speculating that the gene-encoded structures of these proteins may be closely related to their biological functions.

### 3.4. Phylogenetic Relationship and Collinearity Analysis of ABC Gene Family

**Figure 3:**
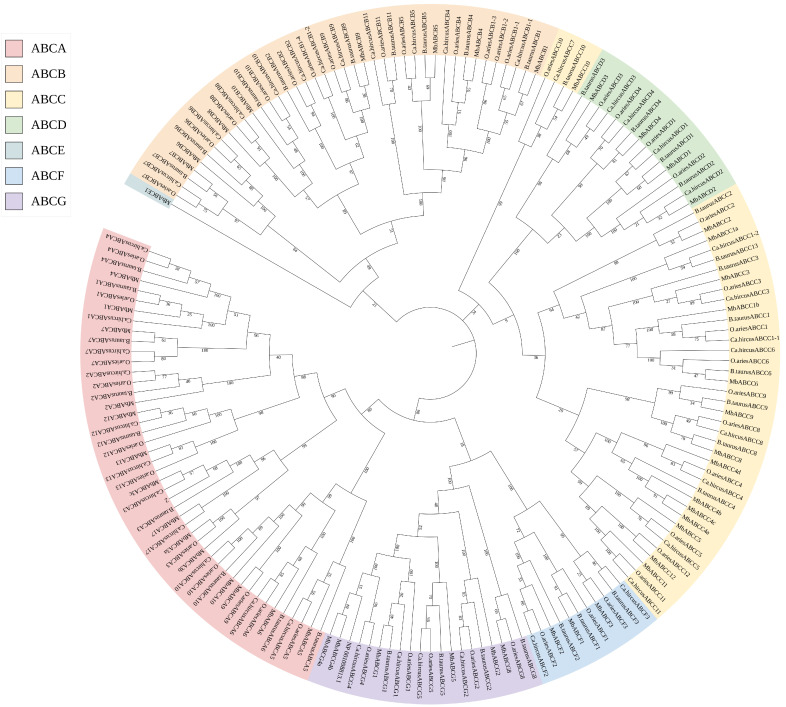
Phylogenetic tree of ABC transporter proteins: A phylogenetic tree was constructed by MEGA12 software with full-length amino acid sequences of FMD, *B. taurus*, *O. aries*, and *Ca. hircus*.

**Figure 4:**
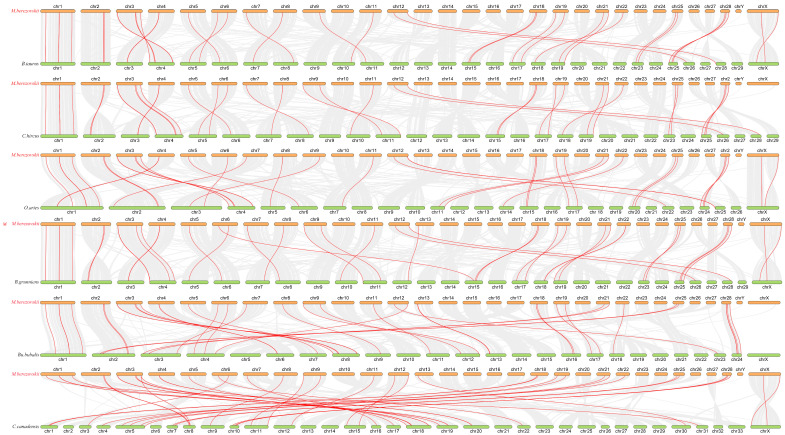
Collinearity analyses of the ABC gene family in FMD, *B. taurus*, *O. aries*, *Ca. hircus*, *Bu. bubalis*, *B. grunniens*, and *Ce. elaphus*. Each horizontal line represents a chromosome, and the numbers represent chromosome numbers. The red lines indicate collinear gene pairs.

It is well known that gene family members are known to originate from a common ancestral gene. Therefore, we selected three mammalian species—*B. taurus*, *O. aries*, and *Ca. hircus* whose ABC gene subfamily classifications are comparable to that of *M. berezovskii*. We then identified *ABC* subfamily protein sequences from each species (Table S5, Figure 3) and constructed pairwise collinearity maps between FMD and six other ruminants: *B. taurus*, *O. aries*, *Ca. hircus*, *Bu. bubalis*, *B. grunniens*, and *Ce. elaphus* (Figure 4).

Phylogenetic analysis revealed that *ABC* genes from FMD consistently clustered closely with their orthologs from the other mammals within each subfamily tree. Notably, genes from the same *ABC* subfamily across different species exhibited closer phylogenetic relationships than genes within the same species, demonstrating subfamily-specific conservation during evolution and indicating higher sequence homology among orthologous subfamily members.

Genome collinearity analysis demonstrated substantial chromosomal correspondence and orthologous conservation between the FMD and *B. taurus*, *O. aries*, *Ca*. *hircus*, *Bu*. *bubalis*, *B*. *grunniens*, and *Ce*. *elaphus* (Figure 4). FMD had 36 collinearities with *B*. *taurus*, 35 collinearities with *O. aries*, and 35 collinearities with *Ca. hircus*, 38 collinearities with *Bu*. *bubalis*, 38 collinearities with *B*. *grunniens*, and 38 collinearities with *Ce. elaphus*. The level of chromosome homology is relatively low, but the level of genome homology is relatively high among these species. In addition, collinearity modules explained the difference in the position of the ABC transporter gene family in FMD relative to the other five species in bovinae and the one species in Cervidae. Most of the ABC genes are distributed on different chromosomes between FMD and other six species, which may be caused by inter-chromosomal rupture or fusion during the evolution. *MbABC12* and *MbABCB6* are distributed on Chr2 with other five species. *MbABCC1a*, *MbABCF3*, *MbABCC5*, and *MbABCG1* are distributed on Chr1, except for *Ce. elaphus*. *MbABCB7* and *MbABCD1* are distributed on ChrX between FMD and *B. taurus*, *O. aries*, *Ca. hircus*, *Bu. bubalis*, and *Ce. elaphus*. However, the lack of collinearity between *MbABCB7* and *MbABCD1* in *Ca. hircus* and *B. grunniens* may have resulted from changes occurring during the evolutionary process.

### 3.5. Gene Structure and Conserved Motif Analysis of MbABC Proteins

**Figure 5:**
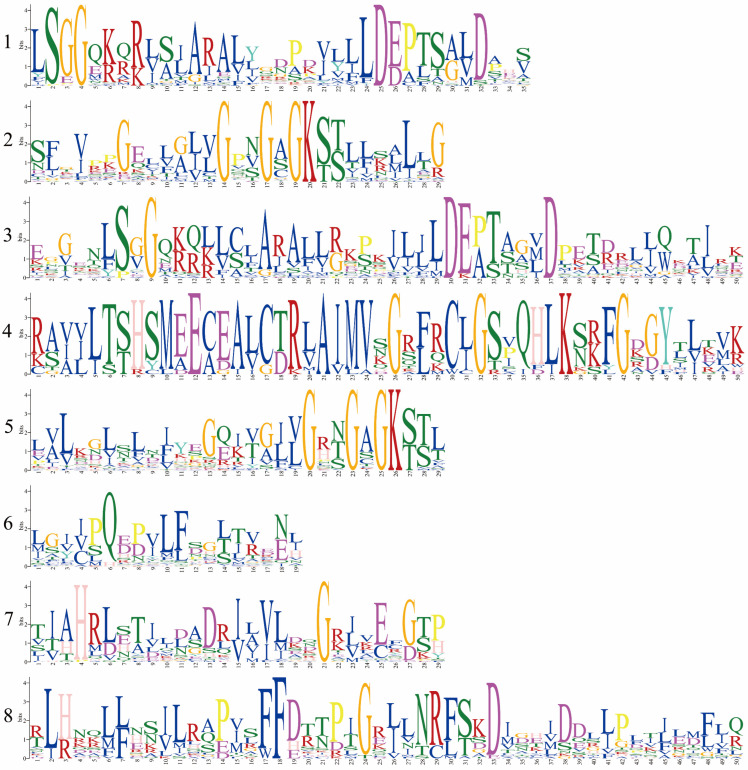
Eight conserved motifs of MbABC proteins.

**Figure 6:**
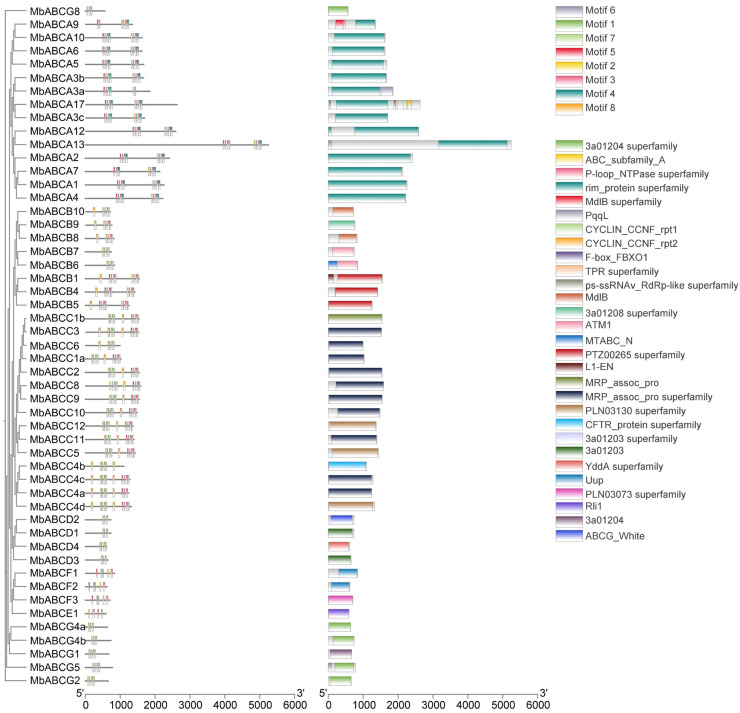
Conserved domain and conserved motifs of MbABC proteins.

**Figure 7:**
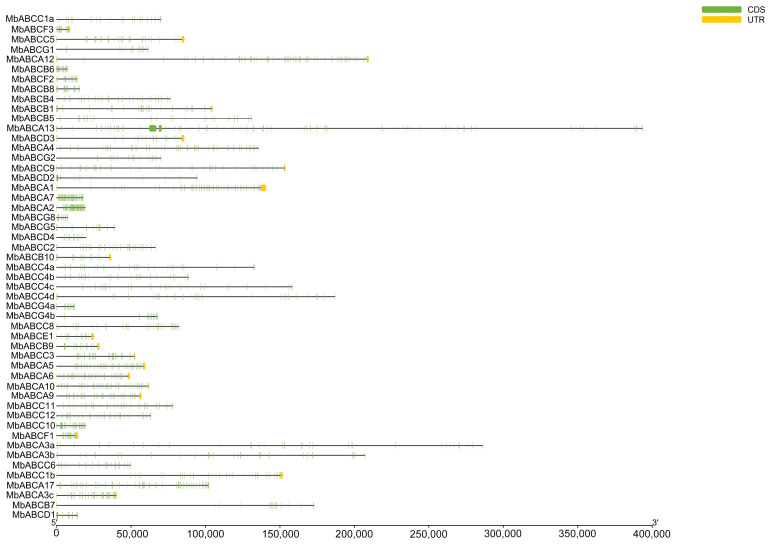
Gene structures analysis of MbABC proteins. Exons and UTR are denoted by green and yellow boxes.

To characterize the putative motifs in MbABC proteins, we submitted the predicted amino acid sequences of the 51 MbABC proteins to the MEME website. The distribution of these motifs in MbABC is shown in Figure 5. To gain insight into the gene structure and evolutionary relationships among members of MbABC proteins (Figure 6 and Figure 7). The motif distribution patterns of MbABC proteins in the same subfamily were similar, indicating that these proteins may have similar functions. The number of conserved motifs in MbABC gene family varied from 3 to 8. Among them, Motif3, motif4, and motif8 was the longest at 50 amino acids, and Motif6, Motif1, Motif7, Motif5, and Motif2 having the highest frequency occurrence across all genes. Motif 6 and 1 were present in each subfamily, with the ABCA and ABCC subfamilies having high number of motifs. Exon–intron structure analysis revealed that MbABC genes have numerous exons. Overall, the organization of probably conserved motifs revealed a correlation with the distinct grouping of ABCs, which may be the cause of the functional specialization of MbABCs in various subfamilies.

### 3.6. KEGG and GO Enrichment Analysis of MbABC Proteins

**Figure 8:**
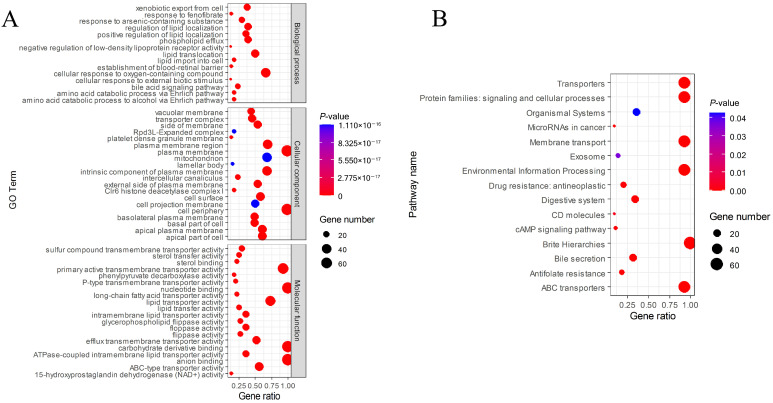
KEGG and GO enrichment analysis of MbABC proteins. (**A**) GO enrichment analysis; (**B**) KEGG enrichment analysis.

Gene Ontology (GO) analysis predicted was further subdivided into 55 sub-categories (Figure 8A). Under the biological process category, the cellular response to oxygen-containing compound subcategory was the most abundant. Among the assignments made to the cellular component category, a large proportion of the sequences were involved in plasma membrane and cell periphery subcategories. In the molecular function category, the majority of the unigene were grouped into nucleotide binding, carbohydrate derivative binding, and anion binding subcategories. KEGG classification indicated that the largest gene ratio is brite hierarchie subgroup, followed by the transports, protein families: signaling and cellular processes, membrane transport, environmental information processing, and ABC transports (Figure 8B).

### 3.7. Protein–Protein Interaction Network Analysis of MbABC Proteins

**Figure 9:**
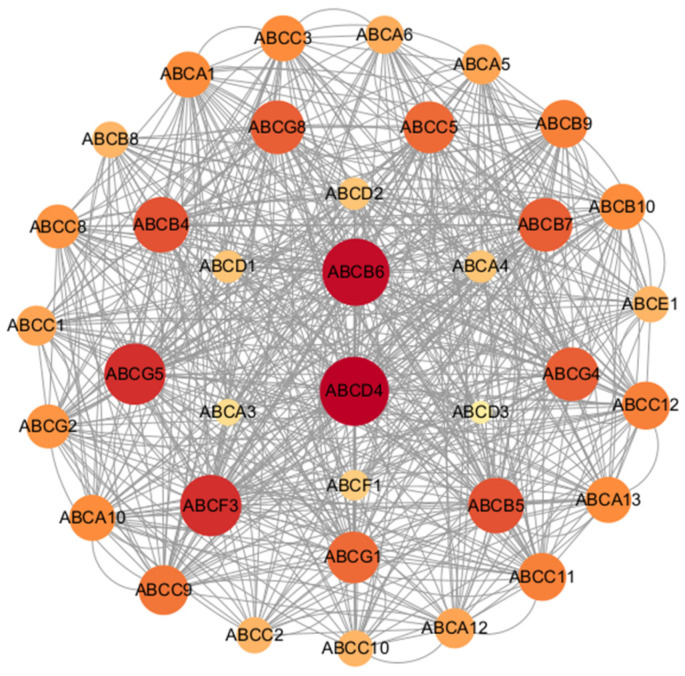
Protein interactions network diagram of MbABC proteins. The protein–protein interaction network of MbABCs: The online webserver STRING was used to annotate the potential MbABCs network, with *M. berezovskii* as input.

We constructed protein–protein interaction (PPI) network for MbABC proteins (Figure 9). Leveraging evidence from text mining, co-expression, homology, and experiments, we constructed a protein interaction network comprised of 36 nodes (proteins) and 598 edges (interactions). Furthermore, protein–protein interactions were also observed between different members of the MbABC family, such as *MbABCD4*, *MbABCB6*, *MbABCF3*, and *MbABCG5*, suggesting potential functional collaborations among *MbABC* proteins. The highly significant PPI enrichment *p*-value (*p* < 0.05) indicates that the network is not random and exhibits strong biological relevance, suggesting the involved proteins collectively participate in a spectrum of related physiological pathways.

### 3.8. Expressive Analysis of the of ABCs in Different Stages and Tissues of FMD

**Figure 10:**
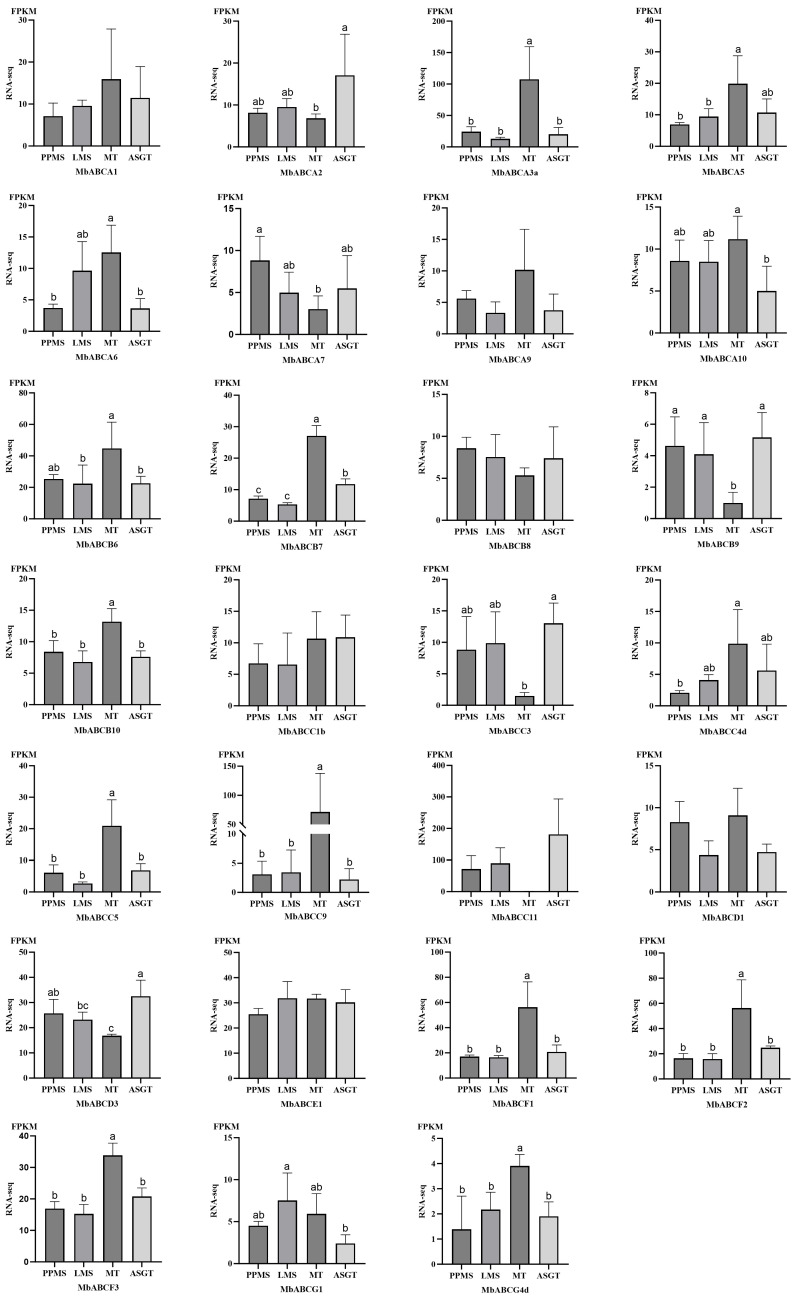
Tissue-specific differential expression of MbABC proteins. Data represent the means ± SE. “a, b, c”, *p* < 0.05.

To investigate the tissue-specific expression of gene members within the ABC transporter family in FMD, we studied the relative expression patterns specifically including incense gland tissue at the peak period of musk secretion (PPMS), the late stage of musk secretion (LMS), muscular tissue (MT), and adolescent gland tissue (MSGT) (Figure 10). They were analyzed and fragments per kilobase of transcript per million mapped reads (FPKM) expression values were used to construct a digital expression level. Results indicate that MbABCA3a, *MbABCA5*, *MbABCB6*, *MbABCB7*, *MbABCC9*, *MbABCC11*, *MbABCF1*, and *MbABCF2* exhibit significant differences, whereas *MbABCA1*, *MbABCA9*, *MbABCB8*, *MbABCC1a*, and *MbABCE1* show no significant differences. During the peak period of musk secretion, *MbABCC11* exhibited the highest relative expression level (71), followed by *MbABCA3a*, *MbABCE1*, *MbABCD3*, and *MbABCB6*, indicating these genes may play crucial roles during musk secretion. During the late musk secretion phase, *MbABCC11*, *MbABCE1*, and *MbABCD3* maintained high relative expression levels. *MbABCA3a* exhibited the highest relative expression in muscle tissue (107), followed by *MbABCC9*, *MbABCF2*, *MbABCB6*, and *MbABCB7*. In conclusion, significant differences exist in the expression of ABC transporter genes across different tissues, suggesting that distinct members may perform unique functions during various stages of musk deer growth and development and in different tissues.

### 3.9. Expression Analysis of MbABC Genes by RT-qPCR

**Figure 11:**
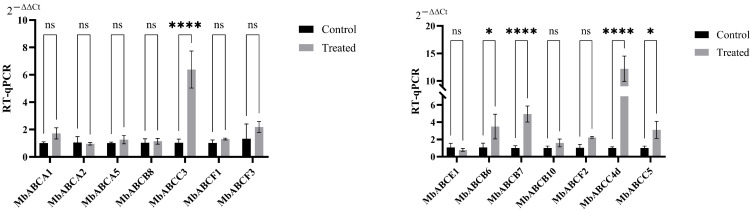
Expression analysis of ABC family genes under abiotic treatment via RT-qPCR in musk gland cells. *, *p* < 0.05; ****, *p* < 0.0001; ns, not significant.

For the non-secreted period (Control) and stimulated (Treated) period of musk gland cells, we conducted RT-qPCR to examine the expression levels of 14 ABC genes (Figure 11). The results revealed that *MbABCA1*, *MbABCA5*, *MbABCB8*, *MbABCB6*, *MbABCB7*, *MbABCB10*, *MbABCC3*, *MbABCC5*, *MbABCF1*, *MbABCF2*, *MbABCF3*, and *MbABCC4d* had expression levels greater in the stimulated period of musk gland cells than that in the non-secreted period of musk gland cells. *MbABCC4d* (~twelve-fold) had the highest expression level in the stimulated period of musk gland cells, followed by *MbABCC3* (~six-fold), *MbABCB7* (~five-fold), *MbABCB6*(~three-fold), and *MbABCC5*(~three-fold). In summary, ABC family members were involved in the stimulated period of musk gland cells and exhibited significant differential expression across different stages, with *MbABCC4d*, *MbABCC3*, *MbABCB7*, *MbABCB6*, and *MbABCC5* potentially playing crucial roles in regulating lipid droplet formation and enhancing muscone content.

## 4. Discussion

The ABC transporter superfamily represents one of the most ancient and evolutionarily conserved protein families, facilitating the ATP-dependent translocation of a diverse array of substrates across biological membranes [41]. With the advent and refinement of high-throughput sequencing technologies, genomic data for an increasing number of species have become available, significantly expanding our capacity to identify and characterize gene families, including ABC transporters, across the tree of life. Their remarkable conservation underscores fundamental cellular roles. To date, comprehensive identification of ABC gene family has been accomplished in various model and non-model organisms, revealing notable variation in family size: *H. sapiens* has 49 ABC genes [32,33], *M. musculus* has 53 ABC genes [42], *Arabidopsis thaliana* has 130 ABC genes [43], *Caenorhabditis elegans* has 55 ABC genes [42]. In the study, we systematical identified 51 ABC transporter genes from the chromosome-level genome of FMD. Phylogenetic classification based on sequence homology and structural architecture has consistently grouped ABC transporters into eight subfamilies (ABCA-ABCH) [11,44]. Subfamilies ABCA through ABCG are ubiquitous in both vertebrates and invertebrates. In contrast, the ABCH subfamily exhibits a more restricted phylogenetic distribution, having been confirmed at the genomic level in teleost fish (e.g., zebrafish, pufferfish), insects (e.g., *Drosophila melanogaster*, *Bombyx mori*) [44,45,46], and the amoeba *Dictyostelium discoideum* [47]. Generally, the ABC transporter complement is relatively conserved among vertebrates but demonstrates considerable diversity within arthropods. For instance, arthropods and mollusks often possess a larger number of ABCG subfamily members compared to vertebrates. While some fish and mollusks contain harbor only a single ABCH, arthropods typically contain multiple. Intriguingly, the citrus mealybug possesses four ABCF genes, whereas three is the typical number in most other animals. Analysis of ABCD, ABCE, and ABCF subfamily numbers in *M. berezovskii*, *Ca. hircus*, *O. aries*, and *B. taurus* (Table S5) revealed a high degree of conservation. Notably, the numbers of ABCG subfamily members in FMD is similar to that in *I. punctatus*, *D. rerio*, and *Oreochromis aureus*, and is higher than in bovid species.

Structurally, ABCA and ABCC subfamilies consist predominantly of full transporters, while ABCD, ABCG, and ABCH are composed of half transporters that must dimerize to form functional units. The ABCB subfamily uniquely contains both half and full transporters. In contrast, ABCE and ABCF subfamilies members contain paired NBDs but lack TMDs, thus lacking transmembrane translocation capacity and are not considered bona fide transporters [48]. However, their NBDs phylogenetically cluster with those of other ABC genes, indicating a conserved evolutionary relationship [17]. Phylogenetic analyses further suggest a close evolutionary relationship between the ABCH and ABCG subfamilies.

Gene duplication events have been pivotal in the expansion and functional diversification of ABC genes. It has been reported that *ABCB1* gene undergone duplication to give rise to the *ABCB4* and *ABCB1* genes in rodents and opossums [9]. The presence of *ABCB1* and *ABCB4* in amphibians and reptiles indicates that these genes likely originated prior to the mammalian-reptilian divergence, a finding that refines previous evolutionary models [9,49]. Subcellular localization predictions for the MbABC proteins indicated that over half are localized to the plasma membrane. Based on sequence characteristics, we classified the MbABC proteins into seven subfamilies: ABCA-ABCG. The PPIs are crucial for understanding complex traits [50]. Our prediction the PPIs network for MbABC proteins, comprising 36 MbABC proteins with 80 nodes in total. Identified *ABCB6* and *ABCD4* as potential hub genes, offering valuable insights for future studies.

The ABC transport genes are often unevenly distributed across chromosomes, a pattern observed in *M. berezovskii*, *C. hanglu yarkandensis*, *I. punctatus*, *D. rerio*, *O. aureus*, and various bovids. This non-uniform distribution is likely a consequence of lineage-specific gene duplication events, such as tandem duplications [51,52]. In *M. berezovskii*, *MbABCC* genes were predominantly located on chromosomes 1, 13, and 22, while the *MbABCG* genes were clustered on chromosomes 9 and 18, suggesting subfamily expansion via gene duplication. We identified several tandemly arrayed gene clusters, including *MbABCG5*/*MbABCG8* on chromosome 9, *MbABCC4a*, *MbABCC4b*, and *MbABCC4c* on chromosome 13, *MbABCG4a*/*MbABCG4b* on chromosome 18, and *MBABCA* clusters on chromosome 21 and 28. *MbABCC1b*/*MbABCC6* on chromosome 28. These tandem repeats appear to be a major mechanism for ABC gene family expansion in the forest musk deer. Furthermore, comparative genomic analysis revealed numerous syntenic blocks of ABC genes between forest musk deer and related artiodactyls (*B. taurus*, *Ca. hircus*, *O. aries*, *Bu. bubalis*, *B. grunniens*, and *Ce. elaphus*), with collinear genes pairs ranging from 35 to 38, highlighting the conservation of genomic context.

ABC transporters facilitate the transport of an exceptionally wide spectrum of substrates, including ions, organic molecules, peptides, complex lipids, and small proteins [53]. In mammals, ABCA subfamily members are critical involved in lipid homeostasis. *ABCA1*, *ABCA2*, *ABCA3*, and *ABCA7* mediate the efflux of cellular cholesterol and phospholipids [54]. Cytochrome transport precursors also depend mainly on the ABCG subfamily [55]. The *ABCA1* proteins are involved in the transport of cholesterol [56,57,58]. The *ABCA4* protein, specific to the photoreceptor cells, transport retinoid–lipid complexes [59,60]. Orthologs of *ABCA1* and *ABCA4* genes were identified in the FMD and three bovine (*B. taurus*, *O. aries*, *Ca. hircus*) genomes. *ABCB1* exhibits broad substrate specificity, effluxing xenobiotics and toxic metabolites [44]. The *ABCB2* (TAP1) and *ABCB3* (TAP2) are transporters associated with antigen processing. *ABCB4* proteins phosphatidylcholine into bile [9], and its absence in bony fish leads to a phospholipid-deficient bile [44]. *ABCB5* gene is highly expressed in melanocytes [9], and *ABCB6* functions as a mitochondrial porphyrin transporter essential for heme biosynthesis [61,62], suggesting a potential detoxification role this gene in moschidae, cervidae, and bovid species. Most ABCC proteins export conjugated compounds and toxins. ABCF members are involved in ribosome assembly and protein translation control [17]. *ABCG1* and *ABCG4* contribute to cellular cholesterol and phospholipids efflux [63], while *ABCG5* and *ABCG8* dimerize to mediate sterols excretion in the intestinal and biliary [18]. The known functions of these subfamilies, particularly in lipid and sterol transport, provide a compelling mechanistic framework for hypothesizing their role in the secretion of musk components, which include numerous lipid-derived and sterol-like compounds.

Tissue-specific expression analysis revealed that during the peak period of musk secretion, the FMD, multiple ABC subfamilies exhibited highly expressed genes, such as *MbABCA3a*, *MbABCB6*, *MbABCC11*, *MbABCD3*, *MbABCE1*, *MbABCF1*, *MbABCF2*, and *MbABCF3*. However, different ABC subfamilies perform distinct functions within cells. We hypothesize that secondary metabolites or other precursor substances generated during the complex biochemical reactions occurring in the FMD during the musk secretion period undergo continuous biochemical transformations within the animal’s body before being ultimately transported to the musk gland to produce musk.

Our expression analysis provides crucial functional insights linking specific MbABC genes to musk secretion physiology. The RT-qPCR revealed that 12 MbABC genes were significantly up-regulated during musk secretion, suggesting potential roles beyond classic transport, possibly in local nutrient or signaling molecule flux. ABC transporters are responsible for transport functions within cells, facilitating the movement of steroids, lipids, secondary metabolites, and other substances. Beijing Yanshe Biotechnology Co., Ltd. (Beijing, China) posits that under agonist stimulation, musk gland cells prematurely enter the secretion phase, producing lipid droplets that are further converted and synthesized into musk. Through RT-qPCR analysis, *MbABCC3* and *MbABCC4d* have up-regulated expression. This suggests that ABC genes may promote the synthesis and transport of lipids or other substances in musk gland cells under these conditions. During musk secretion, genes involved in steroid hormone synthesis play a crucial role and are associated with signal transduction [34,64], and ABCA and ABCC subfamilies participate in this process. Additionally, other ABC subfamily genes may contribute to varying degrees in the musk secretion process of the FMD. These results can provide a reference for researching the response mechanisms of *ABC* genes to the secretion process of FMD. However, the specific molecular mechanisms need to be further studied.

## 5. Conclusions

In summary, this study presents the first genome-wide identification and characterization of the ABC transporter gene family in the FMD. We identified 51 ABC transporter genes, classified them into seven subfamilies (ABCA-ABCG), and mapped their locations across 19 chromosomes. Sequence analysis revealed considerable variation in mRNA length, with the ABCA subfamily being particularly distinct. Physicochemical property analysis indicated that MbABC proteins are generally large, with diverse isoelectric points, lower stability, higher hydrophilicity. These proteins are primarily localized to the plasma membrane and are predominantly composed of α-helices. Tertiary structure models revealed notable similarities between FMD and cattle. Collinearity analysis indicates relatively low levels of chromosomal homology among these species, but relatively high levels of genomic homology. KEGG and GO enrichment analyses confirmed the central role of MbABC genes in transport activities. Subsequent the RT-qPCR validation confirmed that several MbABC genes are up-regulated during the period of active musk secretion. These findings strongly suggest that ABC transporters play a critical role in the musk secretion process, potentially facilitating the maturation and final excretion of musk components. This study provides a valuable foundation for further elucidating the molecular mechanisms of musk secretion and contributes significantly to the scientific basis for the conservation and sustainable utilization of this endangered species.

## Data Availability

The original data presented in the study are openly available in Mendeley Data at https://data.mendeley.com/preview/dvcybyfztv?a=e4a930f8-6adf-4616-873e-c1bae4fa8aff, accessed on 12 December 2025.

## Supplementary Materials

The following supporting information can be downloaded at: https://www.mdpi.com/article/10.3390/ani15243630/s1, File S1: RNA-Seq SOP. Table S1: The primer sequences of MbABC. Table S2: Gene-wide identification of ABC transporter proteins in *M. berezovskii*. Table S3: Protein haracteristics analysis of ABC transporter proteins in *M. berezovskii*. Table S4: Secondary structure and percentage of ABC transporter proteins in *M. berezovskii*. Table S5: The distribution of ABC numbers in FMD, *Bos taurus*, *Capra hircus* and *Ovis aries*. Table S6: Sequence list of the ABC genes identified from *H. sapiens*, *M. musculus*, *B. taurus*, *Ca. hircus*, *O. aries* and *D. rerio* genomes. Refs. [65,66,67,68,69,70,71,72,73,74,75,76,77,78,79,80] are cited in Supplementary Materials.
