# Supplementary material for: Genome-Wide Identification, Function, and Expression Analysis of the ABC Transporter Gene Family in Forest Musk Deer (Moschus berezovskii) Under Musk Secretion Stage
Source: Animals (Basel). 2025 Dec 17;15(24):3630. doi: 10.3390/ani15243630 (PMC12730120; doi:10.3390/ani15243630)
Supplement: Supplementary file 1 [file animals-15-03630-s001.zip › Supplementary Materials File S1.RNA-Seq SOP.pdf]

Transcriptome sequencing

Total RNA isolation and quality control

Total RNA was extracted using the Magen HiPure Universal RNA Mini Kit according to the manufacturer's instructions. Briefly, tissue samples were lysed, and the RNA was bound to a purification column. Following an on-column DNase I digestion to remove genomic DNA, the column was stringently washed to remove impurities. Purified RNA was finally eluted with RNase-free water. RNA quality was rigorously assessed: concentration and purity were measured on a NanoDrop spectrophotometer, with acceptable ratios of A260/A280 (≈1.8–2.1) and A260/A230 (>2.0). Integrity was verified by agarose gel electrophoresis, which showed distinct 28S and 18S ribosomal RNA bands, with the 28S band approximately twice as intense as the 18S band. Only samples passing all quality thresholds were used for subsequent library preparation and sequencing.

Strand-specific library preparation

Following polyA mRNA enrichment using oligo(dT) magnetic beads (Hieff NGS® mRNA Isolation Master Kit, Yeasen, China), the mRNA was fragmented into 200–300 bp pieces by incubation in a magnesium-based fragmentation buffer (Hieff NGS® Ultima Dual-mode RNA Library Prep Kit, Yeasen, China) at 94°C for 5–15 minutes. First-strand cDNA synthesis was performed using these fragmented mRNAs as templates with random hexamer primers and the M-MuLV reverse transcriptase system (Yeasen, China). The RNA strand was then degraded by RNase H (Yeasen, China), and the second strand was synthesised using DNA Polymerase I (Yeasen, China) and dNTPs containing dUTP, thereby incorporating a strand-specific marker. The purified double-stranded cDNA underwent end repair, A-tailing, and ligation to directional adaptors (compatible with Illumina or BGI platforms) using the Hieff NGS® Ultima kit (Yeasen, China). Fragments of approximately 200 bp were size-selected using magnetic bead-based purification modules (Hieff NGS® series, Yeasen, China). Treatment with uracil DNA glycosylase (UDG) selectively degraded the dUTP-containing second strand, preserving only the original first-strand cDNA for strand-specific information. The library was amplified using an ABI 9700 PCR system (Applied Biosystems, USA) and its quality was finally validated with an Agilent DNA 1000 Kit (Agilent, USA).

Transcriptome sequencing

Library quality control was performed prior to sequencing. First, library concentration was quantified using a Qubit 3.0 Fluorometer (Thermo Fisher, USA) with the 1× dsDNA HS Assay Kit (Yeasen, China), requiring a final concentration of ≥2 nM. The size distribution was then analysed on an Agilent 2100 Bioanalyzer (Agilent, USA) with a DNA 1000 Kit to confirm a primary peak in the 350-450 bp range and a DV200 value >80%. Additionally, integrity was verified by 1.5% agarose gel electrophoresis stained with GRred nucleic acid dye (GENEray, China) to ensure the absence of significant smearing or extraneous bands. Libraries meeting all these criteria were deemed suitable for high-throughput sequencing.

Sequencing was performed on either the Illumina or the BGI platform according to experimental requirements. For Illumina sequencing, libraries were loaded onto an S4

flow cell and sequenced using the NovaSeq X Plus Reagent Kit (300 cycles PE150, Illumina, USA), aiming for a cluster density of ≥280 K/mm², a total output of 1-4 Tb per chip, and a Q30 score >85%. For the BGI platform, sequencing was conducted using the DNBSEQ-T7 Sequencing Kit (300 cycles PE150, BGI, China) across four lanes, yielding an output of 6-10 Tb per run. Following sequencing, raw data quality was assessed with FastQC. Reads were then aligned to the reference genome using either STAR or HISAT2 with strand-specificity parameters to preserve the original directional information, resulting in high-quality data for downstream analysis.

Data analysis

Gene expression was quantified as follows. For each individual sample, the high-quality cleaned reads were aligned to the reference genome using HISAT2 (version 2.1.0). The resulting alignment files were used to calculate mapping statistics, including the overall mapping rate. Subsequently, the expression abundance of known transcripts was quantified using StringTie (version 2.1.4). Finally, a unified gene expression matrix across all samples was generated, containing both read counts and FPKM (Fragments Per Kilobase of transcript per Million mapped reads) values.

Gene Enrichment Analysis

Differential gene and transcript expression analysis was performed using DESeq2 (version 1.30) within the R environment (version 4.03). Genes or transcripts with an absolute log2 fold change ≥1 and an adjusted p-value (padj) <0.05 were considered significantly differentially expressed.

These sets of differentially expressed genes (DEGs) and transcripts were then subjected to Gene Ontology (GO) and Kyoto Encyclopedia of Genes and Genomes (KEGG) pathway enrichment analysis using Fisher's exact test.

In addition, Gene Set Enrichment Analysis (GSEA) was conducted to assess the enrichment of predefined gene sets (based on GO terms or KEGG pathways) without applying a hard differential expression cutoff. In this method, all genes were ranked according to their differential expression magnitude between the two sample groups, and a statistical test was employed to determine whether a given gene set was enriched at the top or bottom of this ranked list.

For protein-protein interaction (PPI) network analysis, the STRING database was utilised. Finally, metabolic pathways were visualised at a global level using iPath3.0 to illustrate the distribution within the KEGG metabolic network.

Reference

1. Ashburner, M.; Ball, C.A.; Blake, J.A.; Botstein, D.; Butler, H.; Cherry, J.M.; Davis, A.P.; Dolinski, K.; Dwight, S.S.; Eppig, J.T.; et al. Gene ontology: Tool for the unification of biology. *Nat. Genet.* **2000**, *25*, 25–29. https://doi.org/10.1038/75556.
2. Chen, S.; Zhou, Y.; Chen, Y.; Gu, J. fastp: an ultra-fast all-in-one FASTQ preprocessor. *Bioinformatics* **2018**, *34*, i884–i890. https://doi.org/10.1093/bioinformatics/bty560.
3. Kanehisa, M.; Goto, S. KEGG: Kyoto Encyclopedia of Genes and Genomes. *Nucleic Acids Res.* **2000**, *28*, 27–30. https://doi.org/10.1093/nar/28.1.27.
4. Kim, D.; Langmead, B.; Salzberg, S.L. HISAT: A fast spliced aligner with low memory requirements. *Nat. Methods* **2015**, *12*, 357–360. https://doi.org/10.1038/nmeth.3317.

5. Scholes, A.; Lewis, J.A. Comparison of RNA isolation methods on RNA-Seq: implications for differential expression and meta-analyses. *BMC Genom.* **2020**, *21*, 249. https://doi.org/10.1186/s12864-020-6673-2.
6. Darzi, Y.; Letunic, I.; Bork, P.; Yamada, T. iPath3.0: interactive pathways explorer v3. *Nucleic Acids Res.* **2018**, *46*, W510–W513. https://doi.org/10.1093/nar/gky299.
7. Ura, H.; Togi, S.; Niida, Y. A comparison of mRNA sequencing (RNA-Seq) library preparation methods for transcriptome analysis. *BMC Genom.* **2022**, *23*, 303. https://doi.org/10.1186/s12864-022-08543-3.
8. Love, M.I.; Huber, W.; Anders, S. Moderated estimation of fold change and dispersion for RNA-seq data with DESeq2. *Genome Biol.* **2014**, *15*, 550. https://doi.org/10.1186/s13059-014-0550-8.
9. Pertea, M.; Pertea, G.M.; Antonescu, C.M.; Chang, T.-C.; Mendell, J.T.; Salzberg, S.L. StringTie enables improved reconstruction of a transcriptome from RNA-seq reads. *Nat. Biotechnol.* **2015**, *33*, 290–295. https://doi.org/10.1038/nbt.3122.
10. Modi, A.; Vai, S.; Caramelli, D.; Lari, M. The Illumina Sequencing Protocol and the NovaSeq 6000 System. In *Bacterial Pangenomics*; Methods in Molecular Biology. Humana: New York, NY, USA, 2021; Volume 2242, pp. 15–42. https://doi.org/10.1007/978-1-0716-1099-2_2.
11. Subramanian, A.; Tamayo, P.; Mootha, V.K.; Mukherjee, S.; Ebert, B.L.; Gillette, M.A.; Paulovich, A.; Pomeroy, S.L.; Golub, T.R.; Lander, E.S.; et al. Gene set enrichment analysis: A knowledge-based approach for interpreting genome-wide expression profiles. *Proc. Natl. Acad. Sci. USA* **2005**, *102*, 15545–15550. https://doi.org/10.1073/pnas.0506580102.
12. von Mering, C.; Huynen, M.; Jaeggi, D.; Schmidt, S.; Bork, P.; Snel, B. STRING: A database of predicted functional associations between proteins. *Nucleic Acids Res.* **2003**, *31*, 258–261. https://doi.org/10.1093/nar/gkg034.
13. Soneson, C.; Love, M.I.; Robinson, M.D. Differential analyses for RNA-seq: transcript-level estimates improve gene-level inferences. *F1000Research* **2016**, *4*, 1521. https://doi.org/10.12688/f1000research.7563.2.
14. Liu, X.; Zhao, J.; Xue, L.; Zhao, T.; Ding, W.; Han, Y.; Ye, H. A Comparison of Transcriptome Analysis Methods with Reference Genome. *BMC Genom.* **2022**, *23*, 232. https://doi.org/10.1186/s12864-022-08465-0.
15. Conesa, A.; Madrigal, P.; Tarazona, S.; Gomez-Cabrero, D.; Cervera, A.; McPherson, A.; Szcześniak, M.W.; Gaffney, D.J.; Elo, L.L.; Zhang, X.; et al. A survey of best practices for RNA-seq data analysis. *Genome Biol.* **2016**, *17*, 13. https://doi.org/10.1186/s13059-016-0881-8.
16. Chung, M.; Bruno, V.M.; Rasko, D.A.; Cuomo, C.A.; Muñoz, J.F.; Livny, J.; Shetty, A.C.; Mahurkar, A.; Hotopp, J.C.D. Best practices on the differential expression analysis of multi-species RNA-seq. *Genome Biol.* **2021**, *22*, 121. https://doi.org/10.1186/s13059-021-02337-8.
